# Cloning, characterization, and functional analysis of acetyl-CoA C-acetyltransferase and 3-hydroxy-3-methylglutaryl-CoA synthase genes in *Santalum album*

**DOI:** 10.1038/s41598-020-80268-3

**Published:** 2021-01-13

**Authors:** Meiyun Niu, Haifeng Yan, Yuping Xiong, Yueya Zhang, Xinhua Zhang, Yuan Li, Jaime A. Teixeira da Silva, Guohua Ma

**Affiliations:** 1 Guangdong Provincial Key Laboratory of Applied Botany, South China Botanical Garden, The Chinese Academy of Sciences, Guangzhou, 510650 China; 2 University of Chinese Academy of Sciences, Beijing, 100039 China; 3 Cash Crop Institute of Guangxi Academy of Agricultural Sciences, Nanning, 30007 China; 4 Miki-cho Post Office, Miki-cho, Ikenobe, 3011-2, P.O. Box 7, Kagawa-ken, 761-0799 Japan

**Keywords:** Biotechnology, Molecular biology, Physiology

## Abstract

Sandalwood (*Santalum album* L.) is famous for its unique fragrance derived from the essential oil of heartwood, whose major components are santalols. To understand the mechanism underlying the biosynthesis of santalols, in this study, we cloned two related genes involved in the mevalonate pathway in *S. album* coding for acetyl-CoA C-acetyl transferase (AACT) and 3-hydroxy-3-methyglutary-CoA synthase (HMGS). These genes were characterized and functionally analyzed, and their expression profiles were also assessed. An AACT gene designated as *SaAACT* (GenBank accession No. MH018694) and a HMGS gene designated as *SaHMGS* (GenBank accession No. MH018695) were successfully cloned from *S. album*. The deduced SaAACT and SaHMGS proteins contain 415 and 470 amino acids, and the corresponding size of their open-reading frames is 1538 bp and 1807 bp, respectively. Phylogenetic trees showed that the SaAACT protein had the closest relationship with AACT from *Hevea brasiliensis* and the SaHMGS proteins had the highest homology with HMGS from *Siraitia grosvenorii*. Functional complementation of SaAACT and SaHMGS in a mutant yeast strain deficient in these proteins confirmed that *SaAACT* and *SaHMGS* cDNA encodes functional SaAACT and SaHMGS that mediate mevalonate biosynthesis in yeast. Tissue-specific expression analysis revealed that both genes were constitutively expressed in all examined tissues (roots, sapwood, heartwood, young leaves, mature leaves and shoots) of *S. album*, both genes showing highest expression in roots. After *S. album* seedlings were treated with 100 μM methyl jasmonate, the expression levels of *SaAACT* and *SaHMGS* genes increased, suggesting that these genes were responsive to this elicitor. These studies provide insight that would allow further analysis of the role of genes related to the sandalwood mevalonate pathway in the regulation of biosynthesis of sandalwood terpenoids and a deeper understanding of the molecular mechanism of santalol biosynthesis.

Indian sandalwood (*Santalum album* L.) is a well-studied small hemi-parasitic evergreen tree that is widely distributed in tropical and subtropical regions of India, Indonesia, Australia and the Pacific Islands^1,2^. The scented heartwood of sandalwood and essential oil extracted from it have considerable economic value. Sandalwood is widely used in medicine^3^, wooden handicraft^4^ and cosmetics^5^. The value of sandalwood mainly depends on the proportion of heartwood and on the concentration and quality of essential oil extracted from the heartwood^6,7^. Indian sandalwood is considered to be one of the most valuable sandalwood species, usually yielding 3–8% essential oil^8^. Over 85% of *S. album* essential oil consists of sesquiterpenoids, including sesquiterpene alcohols (α-santalol, β-santalol, *trans*-α-bergamotol and epi-β-santalol) and their corresponding sesquiterpenes (α-santalene, β-santalene, *trans*-α-bergamotene and epi-β-santalene)^8–12^.

Like all other terpenoids, sesquiterpenoids are derived from two building blocks, isopentenyl diphosphate (IPP) and its allylic isomer, dimethylallyl diphosphate (DMAPP). IPP and DMAPP are formed via two pathways. One is the mevalonate (MVA) pathway, which is located in the cytoplasm, and the other is the 2-C-methyl-D-erythritol 4-phosphate (MEP) pathway, which is located in plastids (Fig. 1)^13–16^. The MEP pathway is mainly involved in the biosynthesis of monoterpenoids, diterpenoids and other terpenoids such as hormones, plant pigments, and ubiquitins. The MVA pathway chiefly synthesizes sesquiterpenoids and triterpenoids, for example sterol, as well as other polyterpenoids^17–19^. Since the main components in sandalwood essential oils are sesquiterpenoids, the separation and identification of genes coding for MVA pathway-related enzymes and their functions are essential for further studies of the molecular mechanism of heartwood formation.

**Figure 1:**
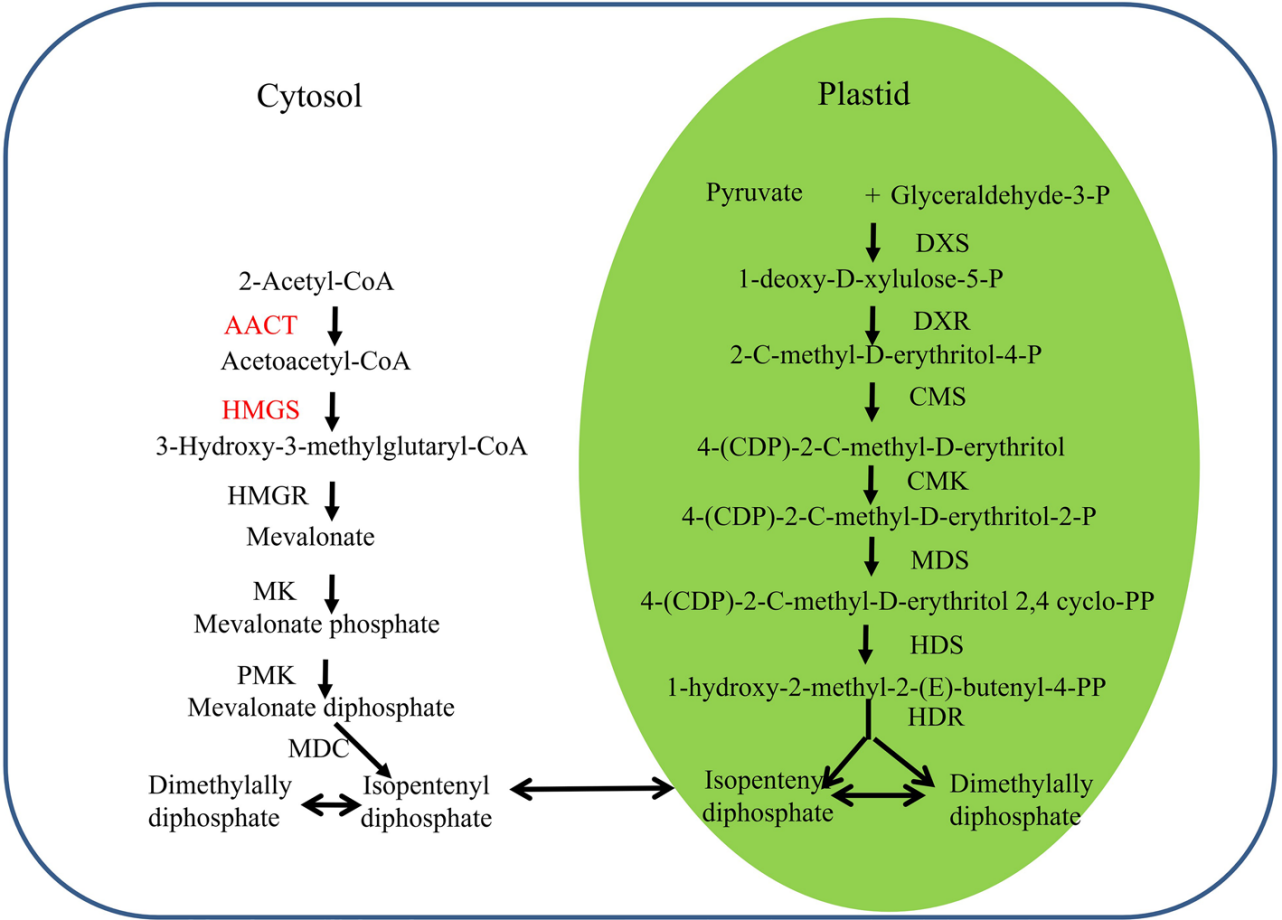
Pathway of isoprenoids biosynthesis in plants (DXS, 1-deoxy-Dxylulose-5-phosphate synthase; DXR, 1-deoxy-D-xylulose-5-phosphate reductoisomerase; CMS, 4-diphosphocytidyl-2-C-methyl-D-erythritol synthase; CMK, 4-diphosphocytidyl-2-C-methyl-D-erythritol kinase; MDS, 2-C-methyl-D-erythritol 2,4-cyclodiphosphate synthase; HDS, 4-hydroxy-3-methylbut-2-enyl diphosphate synthase; HDR, 4-hydroxy-3-methylbut-2-enyl diphosphate reductase). The Figure was drawn by ourselves after referring to previous studies (Laule et al. 2003; Miziorko et al. 2011; Tholl et al. 2015) and tagged the genes marked red were studied.

The MVA pathway generates IPP and DMAPP in six steps, as shown in Fig. 1^20–22^. Acetyl coenzyme A (CoA) acetyltransferase (AACT) catalyzes the condensation of two molecules of acetyl-CoA to form acetoacetyl-CoA through Claisen condensation, and AACT is a regulatory enzyme involved in the biosynthesis of terpenoids under salt stress, promoting the production of squalene^23^. 3-Hydroxy-3-methyglutaryl-CoA synthase (HMGS) transforms acetyl-CoA and acetoacetyl-CoA to 3-hydroxy-3-methylglutaryl-CoA^24^, which is further converted into MVA by 3-hydroxy-3-methylglutaryl-CoA reductase (HMGR). The analysis of HMGS in *S. album* is important to investigate the biosynthesis of α-, β-, epi-β-santalols and bergamotol. In view of their importance in the biosynthesis of terpenoids, the genes related to the MVA pathway have been successfully cloned from a number of plants, such as *Arabidopsis thaliana*^20,25–28,30^, *Salvia miltiorrhiza*^31^, *Aquilaria sinensis*^32^, *Hevea brasiliensis*^33–35^ and *Ginkgo biloba*^36–38^. In contrast, research related to sandalwood terpenoids has mainly focused on terpene synthase, which cyclizes farnesyl pyrophosphate into various cyclic sesquiterpenes such as endo-bergamotene, α-santalene, epi-β-santalene and β-santalene^39–42^, and cytochrome P450 oxygenase, which oxidizes α-, β-, epi-β-santalene and bergamotene to produce α-, β-, epi-β-santalols and bergamotol, respectively^43,44^. To identify the spatial patterns of sesquiterpenoid biosynthesis and to clone genes that encode the enzymes involved in sesquiterpene biosynthesis, transcriptomic analyses of *S. album* have been performed^42^. Srivastava et al. described the cloning and functional characterization of five genes encoding two sesquisabinene synthases (SaSQS1, SaSQS2), bisabolene synthase (SaBS), santalene synthase (SaSS), and farnesyl diphosphate synthase (SaFDS). There are no reports on the upstream genes of the MVA pathway in the biosynthesis of sesquterpenoids in the genus *Santalum*, including *S. album*.

In the present study, we cloned two related genes, named as *SaAACT* and *SaHMGS*, which are involved in the MVA pathway, by RACE technology. This, the first such study for Indian sandalwood, provides more detailed insight into the molecular biology of the santalol biosynthetic pathway. The structure and function of *SaAACT* and *SaHMGS* were analyzed by bioinformatics analyses, functional complementation was performed in yeast, and their expression level in tissues was analyzed by fluorescence quantitative PCR. These studies provide guidance for further analyses of the roles of genes related to the sandalwood MVA pathway in the regulation of biosynthesis of sandalwood terpenoids and a deeper understanding of the molecular mechanism of santalol biosynthesis.

## Results

### Molecular cloning and characterization of the cDNA of *SaAACT* and *SaHMGS*

After sequencing PCR products, it was shown that full-length *SaAACT* is 1538 bp long and contains a 1248 bp open reading frame (ORF) that encodes 415 deduced amino acid residues while full-length *SaHMGS* is 1807 bp long and contains a 1413 bp ORF that encodes 470 deduced amino acid residues. A BLASTn search of *SaAACT* and *SaHMGS* with other plant species showed that both genes are highly homologous to *AACT* and *HMGS* genes from other plant species. *SaAACT* nucleotide sequences showed 80%, 80%, 79%, 79%, and 79% identity with *Camellia oleifera*, *Catharanthus roseus*, *Populus trichocarpa*, *Euphorbia helioscopia* and *H. brasiliensis*, respectively (Table 1). *SaHMGS* nucleotide sequences showed 82% identity with *C. sinensis*, *Panax ginseng*, *H. brasiliensis* and *Siraitia grosvenorii* and 81% similarity with *Platycodon grandiflorus* (Table 1). These results reveal that *SaAACT* and *SaHMGS* belong to the *AACT* and *HMGS* gene families, respectively. Therefore, these genes were designated as *SaAACT* (GenBank accession No. MH018694) and *SaHMGS* (GenBank accession No. MH018695).

**Table 1:** Physicochemical properties of deduced proteins in *Santalum album.*

Proteins	Molecular weight (kD)	Theoretical isoelectric point	Number of acidic amino acids	Number of basic amino acids	Instability index	Aliphatic index	Total average hydropathicity
SaAACT	43.1326	8.71	37	42	32.06	95.01	0.139
SaHMGS	51.9982	6.29	54	50	37.79	75.13	-0.242

### Bioinformatics analysis of the deduced SaAACT and SaHMGS proteins

**Figure 2:**
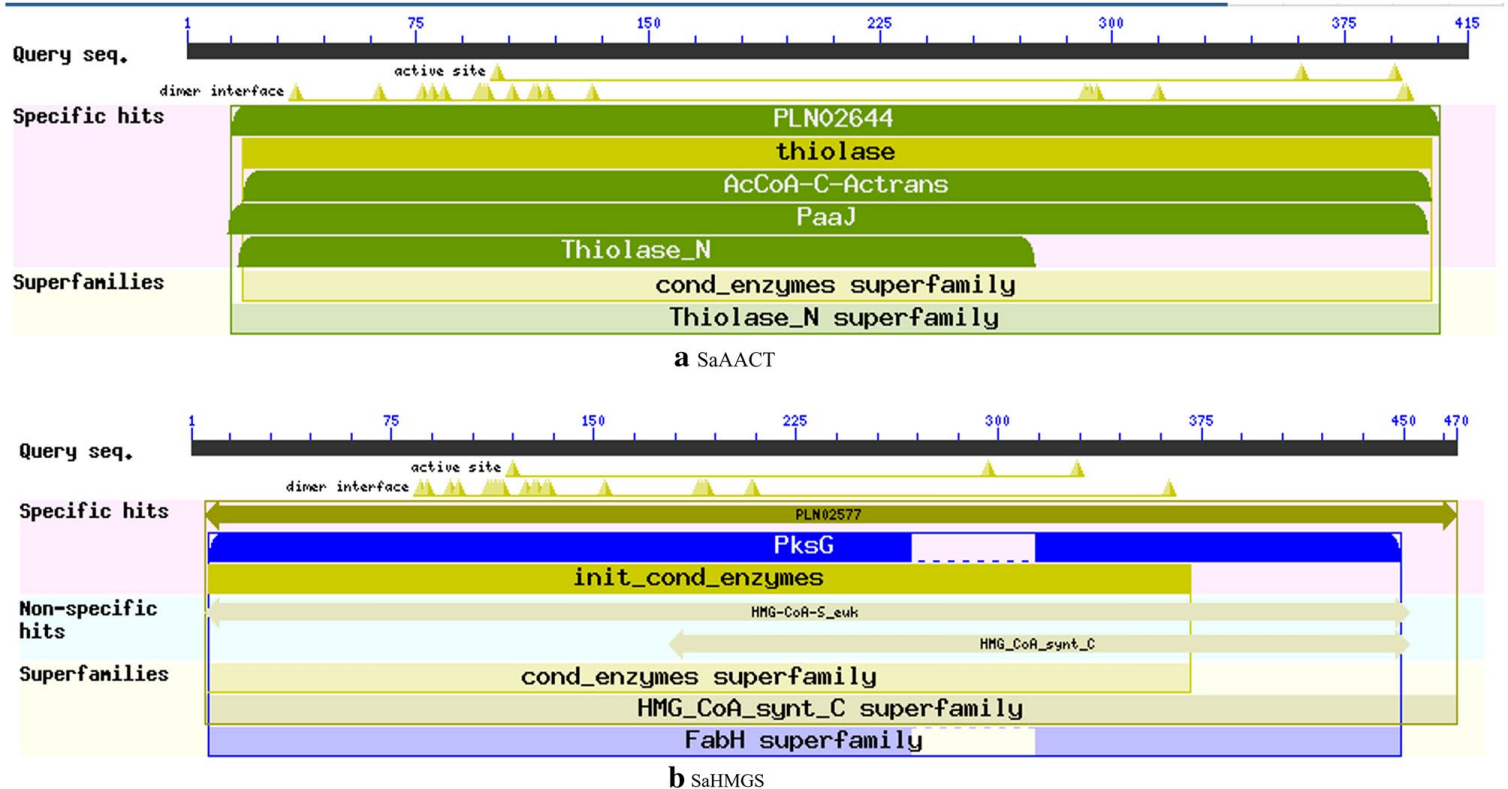
Conserved domain of SaAACT (**a**) and SaHMGS (**b**) in *S. album*. Which was predicted by the Conserved Domains database in NCBI using my own gene sequences.

**Figure 3:**
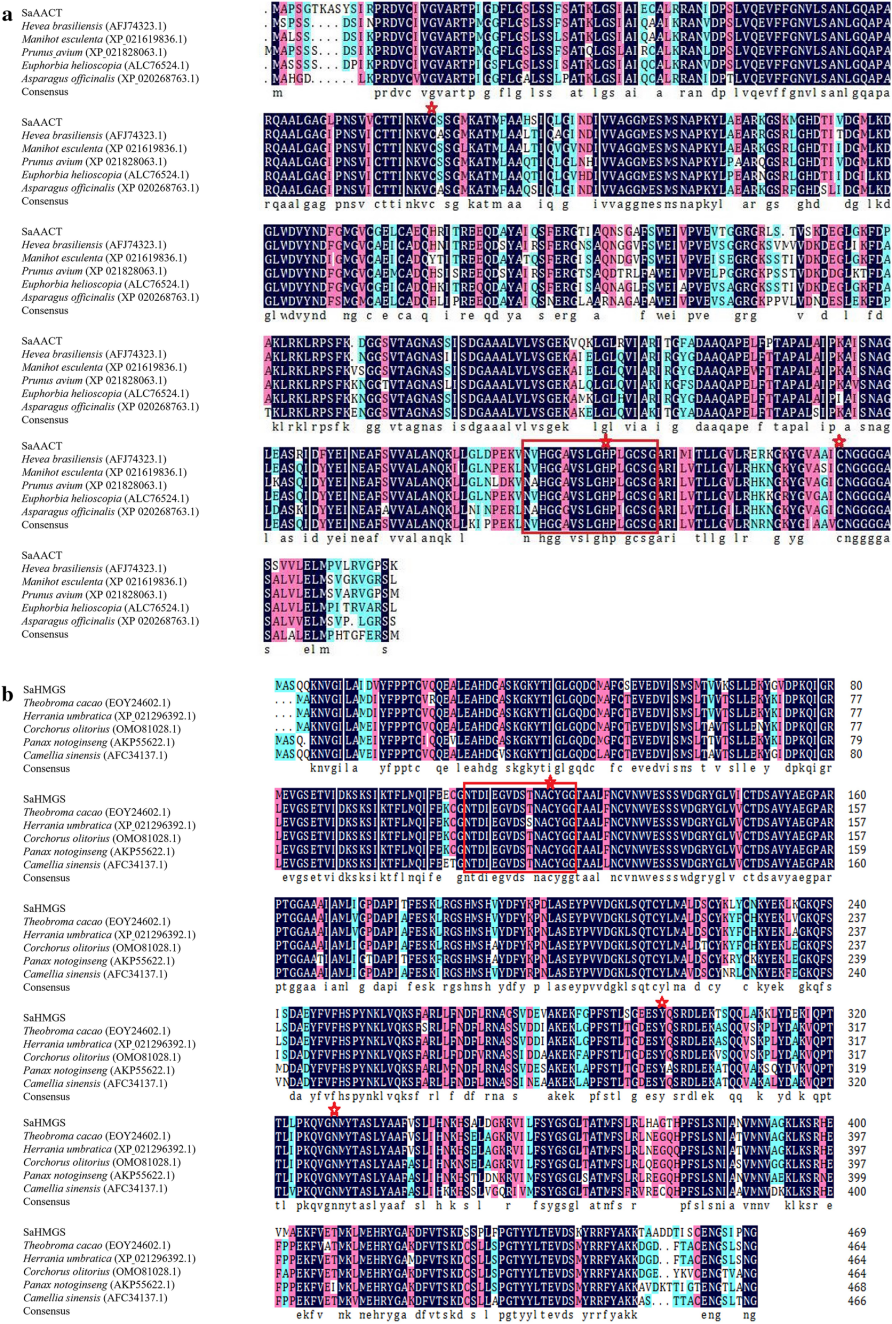
Multiple alignments of SaAACT (**a**) and SaHMGS (**b**) deduced amino acid sequences with other corresponding homologous proteins. The figures were a comparison diagram and cluster analysis of my own gene sequences with related sequences of other species by CLUSTALX 2.0 and MEGA.

The relative molecular weight, theoretical isoelectric point, instability index, aliphatic index and grand average of hydropathicity of the deduced SaAACT and SaHMGS proteins, which were predicted by ExPASy, are shown in Table 2. The relative molecular weight of SaAACT is 43 kDa, similar to previously reported AACT^38^, which had a molecular weight of 41.5 kDa. The relative molecular weight of SaHMGS is 52 kDa, similar to previously reported HMGS^45^, with a molecular weight of 53 kDa. The theoretical isoelectric point of SaAACT and SaHMGS are 8.71 and 6.29, respectively. SaAACT is a stable hydrophobic protein and SaHMGS is a stable hydrophilic protein. Well, SaAACT and SaHMGS have no transmembrane domain or signal peptide. SaAACT has a thiolase (like) domain from aa 19-403 and acetyl-CoA acyltransferase activity from aa 15-406 (Fig. 2a). Residues of two cystines (Cys101, Cys391) and one histidine (His361) are present in SaAACT^46^, and these are highly conserved in AACT among the thiolases from different sources, and are important for its catalytic activity (Fig. 3A, marked with an asterisk). One highly conserved domain (NVHGGAVSIGHPIGCSG) (aa 351-367) at the C-terminal end (Fig. 3a, marked with a red box), which is also present in SaAACT, is a characteristic sequence of thiolase. SaHMGS has significant HMGS activity from aa 4-407 (Fig. 2b). Residues of one cystine (Cys119), one tyrosine (Tyr296) and one aspartic acid (Asn329) are present in SaHMGS^47,48^. They are highly conserved in HMGS from different sources, and are important for its catalytic activity (Fig. 3b, marked with an asterisk). One conserved motif (NxD/NE/VEGI/VDx (2) NACF/YxG) (aa 108-123), which was present in SaHMGS, is a characteristic sequence of HMGS^37,49^. These findings confirm that SaAACT and SaHMGS have similar catalytic functions to the corresponding AACT and HMGS from other plants.

**Table 2:** Nucleotide sequences of target genes and similarity to genes from other plant species.

a: SaAACT		
Species	Accession number	Identity (%)
*Camellia oleifera*	GU594059.1	80
*Catharanthus roseus*	JF739870.1	80
*Populus trichocarpa*	XM002308719.2	79
*Euphorbia helioscopia*	KP995935.1	79
*Hevea brasiliensis*	JN036531.1	78

b: SaHMGS		
Species	Accession number	Identity (%)
*Camellia sinensis*	JQ390224.1	82
*Panax ginseng*	GU565098.1	82
*Hevea brasiliensis*	JN036533.1	82
*Siraitia grosvenorii*	HQ128555.1	82
*Platycodon grandiflorus*	KC439366.1	81

### Phylogenetic analysis of the deduced SaAACT and SaHMGS proteins

**Figure 4:**
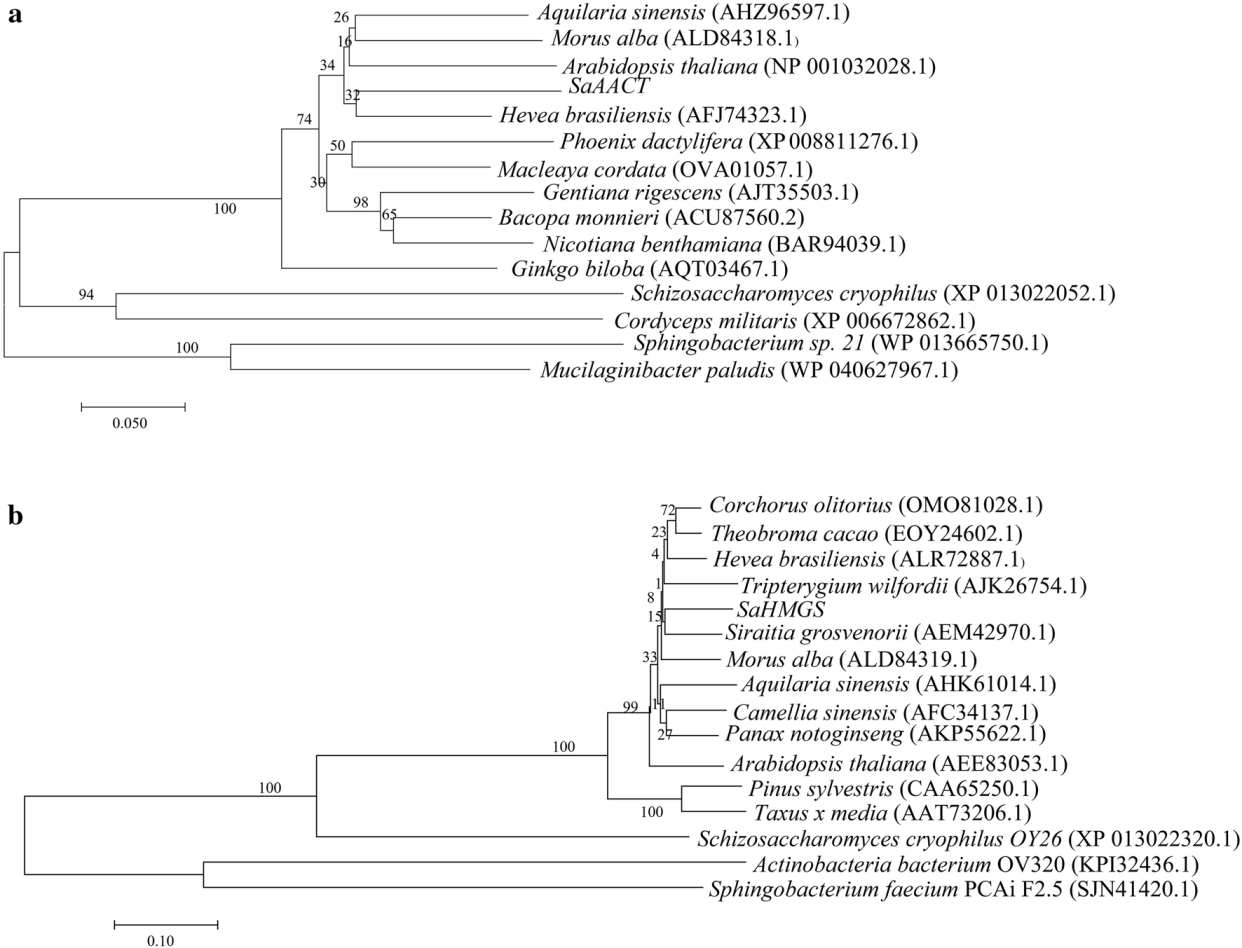
Phylogenetic analysis of target *S. album* proteins (**a**: SaAACT; **b**: SaHMGS).

To better understand the evolutionary relationships between deduced SaAACT and SaHMGS proteins with other AACTs and HMGSs from angiosperms, gymnosperms, fungi, and bacteria, MEGA 7 was used to construct two phylogenetic trees with the neighbor-joining (NJ) method. The first phylogenetic tree revealed that angiosperm AACTs were clustered in one group where SaAACT exhibited the highest homology with AACT from *H. brasiliensis*, while AACT of *G. biloba* formed a distinct group of gymnosperms, and the AACT gene from fungi and bacteria were clustered as a different group (Fig. 4a). The second phylogenetic tree indicated that angiosperm HMGSs were clustered in one group where SaHMGS exhibited the closest homology with HMGS from *S. grosvenorii*, while HMGS of *Pinus sylvestris* and *Taxus* × *media* formed a distinct group of gymnosperms and the *AACT* gene from fungi and bacteria were clustered as a separate group (Fig. 4b). These results suggest that SaAACT and SaHMGS share a common evolutionary base with other plant AACT and HMGS proteins based on their conserved structure and sequence characteristics.

### Sub-cellular localization of SaAACT and SaHMGS proteins

**Figure 5:**
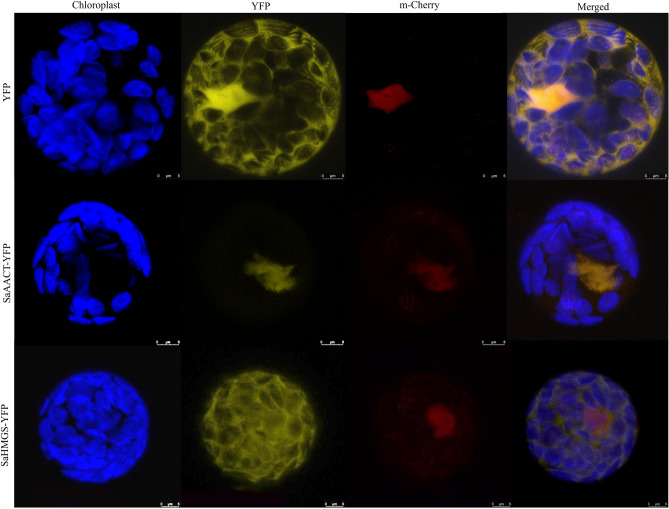
Subcellular localization of SaAACT and SaHMGS proteins in *A. thaliana* mesophyll protoplasts. Yellow fluorescence indicates SaAACT-YFP and SaHMGS-YFP fusion protein signal. Blue signal indicates chlorophyll (Chl) autofluorescence and red signal indicates m-Cherry fluorescence. The merged images represent a digital combination of Chl autofluorescence, YFP fluorescent and m-Cherry protein fluorescence images. Fluorescence was excited for YFP at 514 nm, for Chl at 543 nm and for m-Cherry at 587 nm. Scale bar of SaHMGS-YFP and YFP = 5 μm and scale bar of SaAACT-YFP = 8 μm.

Predicted sub-cellular localization of SaAACT and SaHMGS proteins by PSORT showed that SaAACT has the highest probability scores for peroxisomes (0.78), followed by the cytosol (0.11) and mitochondria (0.11) whereas SaHMGS exists mainly in the cytosol (0.61) but is also distributed in the nucleus (0.22) and mitochondria (0.17). To further verify the sub-cellular localization of SaAACT and SaHMGS protein, sub-cellular localization of SaAACT-YFP (yellow fluorescent protein) and SaHMGS-YFP were studied using a modified polyethylene glycol method to transform *Arabidopsis thaliana* protoplasts with SaAACT-YFP and SaHMGS-YFP constructs. In *A. thaliana*, AACT2, which is involved in the MVA pathway, is localized in the cytosol and nucleus whereas AACT1, which may be involved in fatty acid degradation, is located in peroxisomes^50,51^. We found that SaAACT proteins were located in the nucleus (Fig. 5). Sub-cellular localization of SaHMGS-YFP showed that SaHMGS proteins were located in the cytosol (Fig. 5), like BjHMGS1 of *Brassica juncea*^52^. Our results suggest that SaAACT and SaHMGS cloned in this study may be involved in MVA pathway in *S. album*.

### Functional complementation of *SaAACT* and *SaHMGS* in yeast

**Figure 6:**
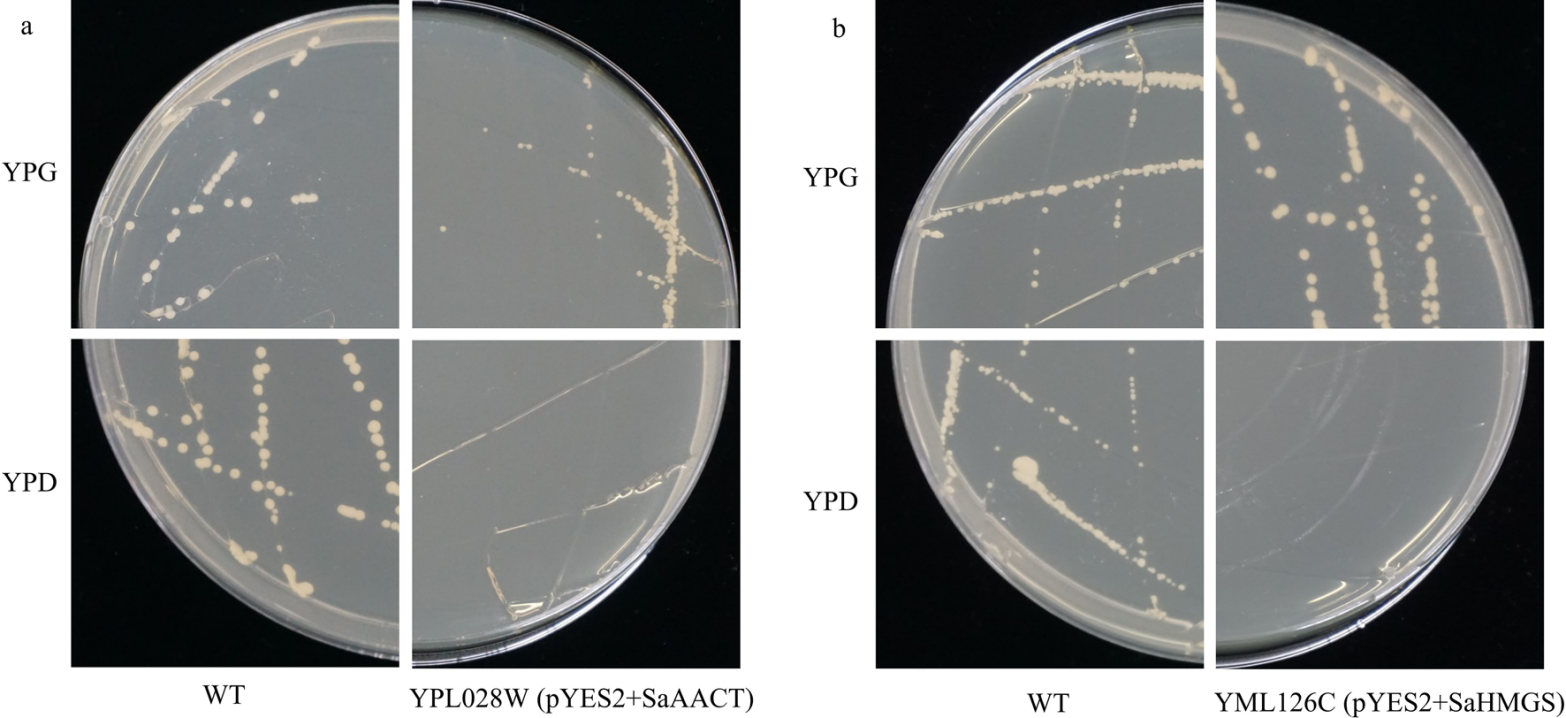
Functional complementation of two *Santalum album* genes (**a**: *SaAACT*; **b**: *SaHMGS*) in yeast (*Saccharomyces cerevisiae*). WT, wild type.

In yeast, the MVA pathway is a biosynthetic pathway that is essential for survival, and disrupting MVA pathway genes in yeast strains can be fatal^53,54^. The disrupted strains with empty pYES2 could not grow on either YPG expression medium or YPD non-expression medium (Fig. 6a). YPL028W harbored pYES2-*SaAACT* and YML126C harbored pYES2-*SaHMGS*, which grew well on YPG medium. However, neither YPL028W, which harbored pYES2-*SaAACT*, nor YML126C, which harbored pYES2-*SaHMGS*, could grow on YPD medium (Fig. 6b). These results indicate that *SaAACT* and *SaHMGS* have AACT and HMGS activity, respectively.

### Tissue-specific and MeJA treatment expression analysis of *SaAACT* and *SaHMGS*

**Figure 7:**
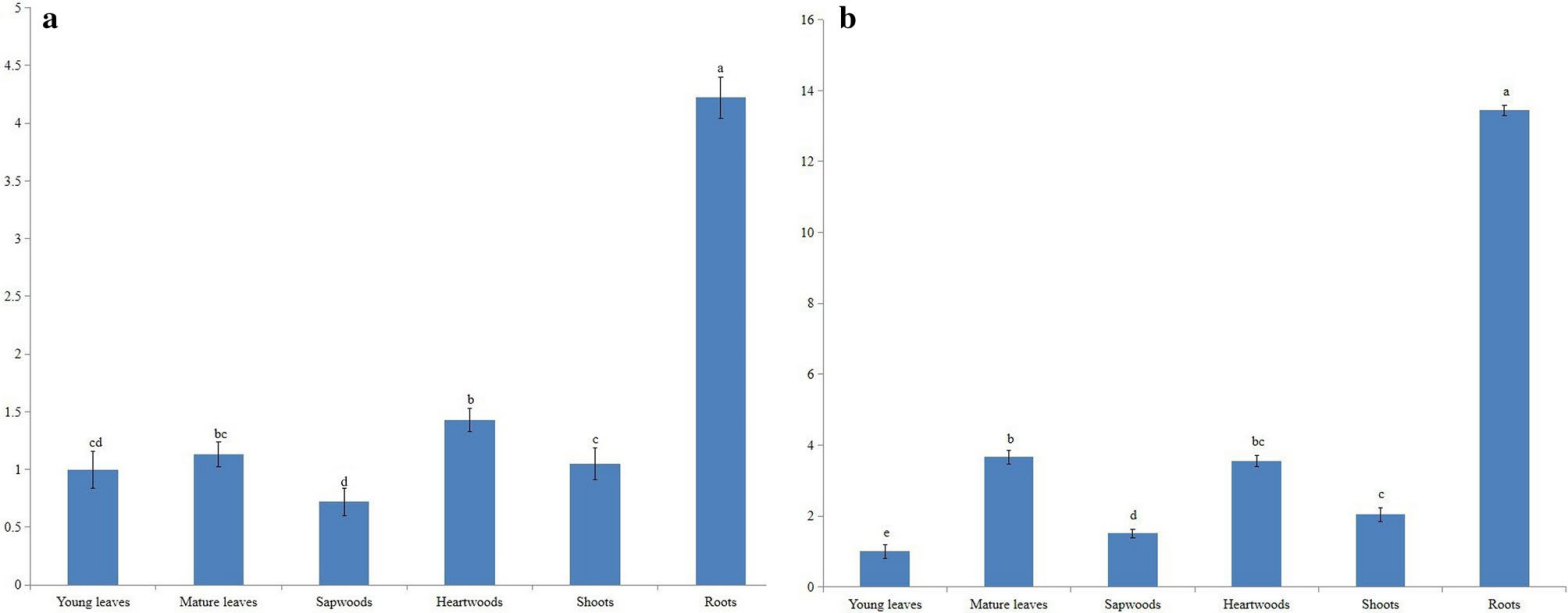
Gene expression levels of *SaAACT* (**a**) and *SaHMGS* (**b**) in different organs of *Santalum album* using qRT-PCR. The gene expression level of *SaAACT* and *SaHMGS* in the young leaves was set to 1. Data from qRT-PCR are means ± SD (standard deviation) from triplicate experiments (n = 3). Different letters indicate significant differences (*p* < 0.05) following one-way ANOVA, using Duncan’s multiple range test.

To explore the tissue-specific expression pattern of *SaAACT* and *SaHMGS* genes in *S. album*, total RNA was isolated from different tissues, including roots, heartwood, sapwood, young leaves, mature leaves and shoots. quantitative real-time PCR (qRT-PCR) was also performed. The results of qRT-PCR are shown in Fig. 7. *SaAACT* and *SaHMGS* were constitutively expressed in all tissues*.* The *SaAACT* gene was differentially expressed in various tissues and exhibited the highest level of expression in roots, followed by mature leaves and heartwood, approximately 13.44-, 3.66- and 3.55-fold higher than young leaves, respectively. A similar expression pattern of the *AACT* gene was found in *Bacopa monnieri* and *G. biloba*, in which the *BmAACT* and *GbAACT* genes were highly expressed in roots, followed by stems and leaves^38,55^. As shown in Fig. 7, *SaHMGS* was highly expressed in roots, approximately 4.22-fold more than in young leaves. The expression level of *SaHMGS* in mature leaves, sapwood, heartwood, shoots and young leaves showed few differences. In contrast, in *Taxus* × *media*, *TmHMGS* was expressed in needles and stems at a similar level, but no expression was detected in roots^45^.

**Figure 8:**
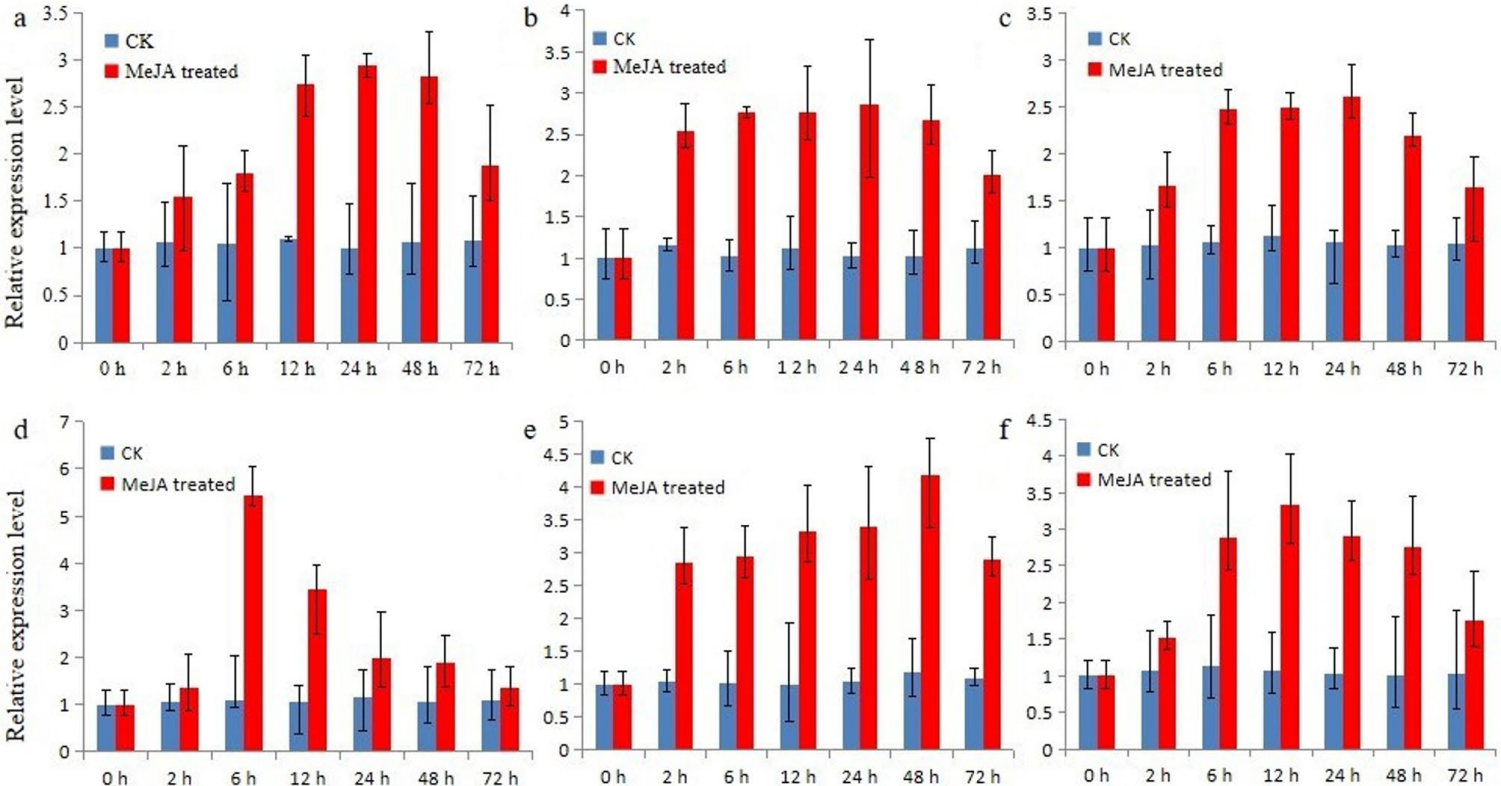
Level of *SaAACT* (**a**,**b**,**c**) and *SaHMGS* (**d**,**e**,**f**) transcripts in *Santalum album* roots (**a**,**d**), shoots (**b**,**e**) and leaves (**c**,**f**) after induction by 100 mM MeJA. The expression level of *SaAACT* and *SaHMGS* genes in the untreated control (CK) was set to 1. Data from qRT-PCR are means ± SD (standard deviation) from triplicate experiments (n = 3).

Methyl jasmonate (MeJA) is a small signaling molecule that can regulate secondary metabolism in plants when applied exogenously^56^. MeJA is closely related to terpene metabolism^57^. MeJA treatment can effectively induce the expression of *AACT* and *HMGS* genes and terpenoid biosynthesis in *S. miltiorrhiza*^58^, *G. biloba*^38^ and *Tripterygium wilfordii*^59^. In this study, we measured the transcript level of *SaAACT* and *SaHMGS* in *S. album* roots, shoots and leaves after treatment with 100 μM MeJA (Fig. 8). *SaAACT* and *SaHMGS* expression were significantly induced by MeJA. The trend of the change in transcript level of *SaAACT* in *S. album* roots, shoots and leaves after MeJA treatment was consistent, all increasing gradually and peaking at 24 h compared with control seedlings in which *SaAACT* transcript level decreased slowly after MeJA treatment. With regard to the *SaHMGS* gene, the trends in the change of transcript level of *SaAACT* in *S. album* roots, shoots and leaves after MeJA treatment were different. In roots, expression level increased rapidly after seedlings were treated with MeJA for 6 h, then gradually decreased. In shoots, expression increased slowly then peaked at 48 h after MeJA treatment. In leaves, expression level gradually increased and peaked at 12 h after MeJA treatment, then gradually decreased.

## Discussion

The commercial value of sandalwood lies mainly in its fragrant heartwood. The final purpose of planting sandalwood is to harvest high quality heartwood in great quantities. However, slow growth rates, susceptibility to diseases and variation in sandalwood oil yield hamper sandalwood production^43^. Researchers have attempted to synthesize santalol by chemical approaches^60–62^, but yield is very low and industrial production is not economic. In 1998, sandalwood was listed on the red list of threatened plants as a vulnerable plant by the International Union for Conservation Nature and Natural Resources^63^. Currently there are no reports for *S. album* on the genes upstream of the MVA pathway involved in sesquiterpenoid biosynthesis.

This study is the first to clone and characterize the *SaAACT* and *SaHMGS* genes encoding AACT and HMGS of the MVA pathway and analyze their tissue expression levels. A 1538 bp full-length cDNA of the *SaAACT* gene was isolated from *S. album*. The deduced SaAACT protein contained 415 amino acids and weighed 43 kDa. In addition, an 1807 bp full-length cDNA of the *SaHMGS* gene was isolated from *S. album*. The deduced SaHMGS protein contained 470 amino acids and weighed 52 kDa. Bioinformatics analysis showed that *SaAACT* and *SaHMGS* genes have high similarity with other corresponding genes in other plants. The deduced amino acids of SaAACT and SaHMGS have typical motifs and domains of AACT and HMGS proteins. Multiple alignments and phylogenetic analyses showed that SaAACT and *H. brasiliensis* AACT were located in the same clade, while SaHMGS and *S. grosvenorii* HMGS were clustered together in a separate clade, indicating a close genetic relationship between these protein pairs. These results suggest that SaAACT and SaHMGS are conserved across different plants based on their conserved structure and sequence characteristics. Functional complementation experiments in yeast showed that *SaAACT* and *SaHMGS* encode functional AACT and HMGS, respectively. The expression of *HMGS* genes is related to the accumulation of terpenoids^64^. Overexpression of *AACT* and *HMGS* genes can increase the level of sesquiterpenoids in many plants. Previous studies showed that the expression patterns of *AACT* and *HMGS* genes vary considerably in different plants^50,60^. The qRT-PCR results showed that *SaAACT* and *SaHMGS* genes were constitutively expressed in all the tested tissues but were differentially expressed in various tissues. The transcript level of *SaAACT* in roots was higher than in other tissues followed by mature leaves and heartwood, and the lowest expression level was in young leaves, which is consistent with a previous study^55^. The transcript level of *SaHMGS* in roots was higher than in other tissues, and the lowest expression level was in sapwood, which contrasts to *SmHMGS* reported previously^45,58^. Our results revealed that *SaAACT* and *SaHMGS* were expressed in all tissues, but at higher levels in roots. Many studies have shown that the yield of terpenoids is positively correlated with the expression of *AACT* and *HMGS* genes. Previous studies demonstrated that the accumulation of terpenoids increased in *AACT* overexpressing transgenic plants^65,66^. In *Ganoderma lucidum*, triterpene content increased in Gl-aact overexpressing transformants^65^. Overexpression of *A. thaliana AACT* in *Taraxacum brevicorniculatum* latex increased sterol levels by about five-fold^66^. An *Escherichia coli* strain that was transformed with a codon-optimized *HMGS* gene exhibited significantly more bisabolene production than control bacteria^67^. *BjHMGS* overexpressing transgenic plants significantly increased the total sterol content in leaves and seedlings^68^. Our results revealed that the expression levels of *SaAACT* and *SaHMGS* were highest in roots followed by heartwood. This trend is consistent with the chemical composition analysis in earlier reports^69,70^, which showed that roots and heartwood are used to extract essential oils. These results support the relationship between transcription levels of *SaAACT* and *SaHMGS* and the content of sesquiterpenoids, indicating that *SaAACT* and *SaHMGS* may play an important role in the production of sesquiterpenoids in *S. album*. Our results show that the transcript levels of *SaAACT* and *SaHMGS* in roots, shoots and leaves of *S. album* seedlings increased after MeJA treatment, implying that the putative *SaAACT* and *SaHMGS* were responsive to the elicitor, MeJA, and could be induced, at least at the transcriptional level. Studying the biosynthesis of terpenoids at the molecular level is an important way to understand the mechanism of heartwood formation. The expression profiles of *AACT* and *HMGS* genes, which code for key enzymes in the biosynthesis of sesquiterpenes in plants, suggest that MeJA treatment may be an effective way to induce a high yield of sesquiterpenes in *S. album*. However, the regulatory mechanism of *AACT* and *HMGS* genes in the biosynthesis of sesquiterpenes in *S. album* needs to be clarified through additional studies.

The findings of our study are not only helpful to further understand the biosynthesis of santalols, but also provide a theoretical basis for further studies on the prokaryotic expression of related proteins. This study provides a molecular resource for increasing the content of santalols by genetic engineering.

## Materials and methods

Young leaves of five-year-old sandalwood trees (*S. album*) growing in South China Botanical Garden, Guangzhou, were used to isolate the *SaAACT* and *SaHMGS* genes. The young and mature leaves, shoots, heartwood, sapwood and roots were harvested to test the tissue-specific expression of *SaAACT* and *SaHMGS* genes by qRT-PCR. All collected samples were wrapped in tin foil, frozen immediately in liquid nitrogen, and then stored at −70 °C until further use.

### Total RNA extraction from sandalwood leaves and first-strand cDNA synthesis

Total RNA of sandalwood leaves was extracted using Column Plant RNAOUT (Tiandz, Beijing, China) according to the manufacturer’s protocol. The quantity and quality of isolated RNA was measured with a NanoDrop ND-1000 spectrophotometer (Nanodrop Technologies, Wilmington, NC, USA). The integrity of isolated RNA was detected on a 1% agarose gel. High quality RNA was stored in DEPC-treated water at – 70 °C for future use. First strand cDNA were obtained with the PrimeScript first-strand cDNA synthesis kit (Takara Bio Inc., Dalian, China). Synthesized cDNA was stored at − 20 °C and served as the template for downstream reactions. For qRT-PCR, total RNA was isolated from different tissues (i.e.: roots, heartwood, sapwood, young leaves, mature leaves, and shoots) and first strand cDNA was synthesized as described above.

### Isolation and cloning of full-length *SaAACT* and *SaHMGS* cDNA by RACE

The full-length cDNA sequence of *SaAACT* and *SaHMGS* genes was isolated by 5′ and 3′ rapid amplification of cDNA ends (RACE)-PCR with the SMARTer RACE cDNA Amplification Kit (Clontech, Palo Alto, CA, USA) according to the manufacturer’s protocol. Gene-specific primers for 3′ RACE-PCR were designed on the basis of the initial data of AACT and HMGS unigenes in the *S. album* transcriptome^71^. Based on data from the partial *SaAACT* and *SaHMGS* sequence generated from 3′ RACE-PCR, gene-specific primers were designed for 5′ RACE-PCR to obtain the remaining sequences of *SaAACT* and *SaHMGS*. PCR products were purified by gel DNA purification kits (Tiangen, Beijing, China) and ligated into the pMD18-T vector (Takara Bio Inc., Dalian, China). Recombined plasmids were transformed into DH5α-competent *E. coli* cells (Takara Bio Inc., Dalian, China) and sequenced.

### Bioinformatics analysis of *SaAACT* and *SaHMGS*

The nucleotide and amino acid sequences of *SaAACT* and *SaHMGS* were analyzed by bioinformatics methods, and their physical and chemical characteristics, transmembrane domain, signal peptide, and subcellular localization were predicted by corresponding bioinformatics software. Sequence assembly was performed with DNAstar (http://www.dnastar.com). Nucleotide sequences, deduced amino acid sequences and ORFs were analyzed, and sequences were compared through a BLAST database search (http://www.ncbi.nlm.nih.gov). Protein molecular weight and theoretical isoelectric point, instability index, aliphatic index and grand average of hydropathicity were calculated by ExPASy (http://www.expasy.ch/tools/). Protein conserved domains and active sites were predicted by the Conserved Domains database in NCBI (http://www.ncbi.mlm.nih.gov/Structure/cdd/wrpsb.cgi) and by Prosit (http://prosite.expasy.org/) in ExPASy. Transmembrane domains were analyzed on the TMHMM Server (http://www.cbs.-dtu.dk/services/TMHMM/). Signal peptides were analyzed by SignalP (http://www.cbs.dtu.dk/services/SignalP/). Protein subcellular localization was predicted by PSORT II (http://psort.org/).

### Molecular evolution analyses of SaAACT and SaHMGS proteins

A phylogenetic tree of SaAACT and SaHMGS proteins from *S. album* and other plants, including angiosperms, gymnosperms, fungi, and bacteria, was constructed by the NJ method^72^ with CLUSTALX 2.0 (Conway Institute, University College Dublin, Dublin, Ireland) and MEGA 7^73^. The calculation of evolutionary distance was based on the Poisson correction method with 1000 bootstrap repeats.

### Subcellular localization of SaAACT and SaHMGS

A vector pSAT6-EYFP containing the ORF of enhanced yellow fluorescent protein (EYFP) was used in this study. The cDNA encoding *SaAACT* and *SaHMGS* were amplified with two pairs of primers, YFP-AACT-F and YFP-AACT-R, and YFP-HMGS-F and YFP-HMGS-R, respectively (Table 3). The PCR products were digested by *EcoR* I and *BamH* I restriction enzymes. The digested fragment was ligated into *EcoR* I- and *BamH* I-digested pSAT6-EYFP vector to generate pSAT6-EYFP-SaAACT and pSAT6-EYFP-SaHMGS fusion constructs. The fusion expression vectors and the pSAT6-EYFP vector were transformed into *A. thaliana* mesophyll protoplasts followed a method described previously^74^. After 16–24 h of incubation at 22 °C, YFP fluorescence in transformed protoplasts of *A. thaliana* was observed using a confocal laser-scanning microscope (Leica TCS SP8 STED 3X, Wetzlar, Germany).

**Table 3:** Specific primers used in this study.

Primer purpose	Primer name	Primer sequence (5′ → 3′)
5′ RACE	AACT-5(1)/(2)	CACAAACACCCATGCCAAAATCA/TGCTTTCCATGCCACCAGCTACA
	HMGS-5(1)/(2)	AGTCTCGCTGCCTACTTCCATCC/TCCAGTGCTTCCTGTTGAACACA
3′ RACE	AACT-3(1)/(2)	GAGGAAGCTCCGACCAAGTTTTA/GGAGCTGTATCTCTGGGACATC
	HMGS-3(1)/(2)	CTCAGTCAGCATGCCTAAACCT/CATTGTTGCCGGCTCTGTTC
ORF	AACT-O(F)/(R)	ATGGCTCCATCCGGGACGAAAGC/TAGCTTTGAAGGTCCTACTCTCA
	HMGS-O(F)/(R)	ATGGCTTCGCAGCAGAAGAAT/TTAATGGCCATTGGGAATTGAACCA
qRT-PCR	q-AACT-F/R	CGGGACGAAAGCCTCTTATT/AGGGAACCAAGAAAGTCACC
	q-HMGS-F/R	TCTCGCCATTGATGTCTACTTT/CCCAATGGTGTACTTCCCTTTA
Functional complementation	pYES2-AACT-F	CAGTGTGCTGGAATTCATGGCTCCATCCGGGACG
	pYES2-AACT-R	CATGCTCGAGCGGCCGCTAGCTTTGAAGGTCCTACTCTCAAG
	pYES2-HMGS-F	CAGTGTGCTGGAATTCATGGCTTCGCAGCAGAAGA
	pYES2-HMGS-R	CATGCTCGAGCGGCCGCATGGCCATTGGGAATTGAACCA
Subcellular localization	YFP-AACT-F	CCGGAATTCATGGCTCCATCCGGGACGAAAGC
	YFP-AACT-R	CGCGGATCCTAGCTTTGAAGGTCCTACTCTCA
	YFP-HMGS-F	CCGGAATTCATGGCTTCGCAGCAGAAGAA
	YFP-HMGS-R	CGCGGATCCATGGCCATTGGGAATTGAACCA

### Functional complementation of *SaAACT* and *SaHMGS* in yeast

To determine the function of *SaAACT* and *SaHMGS*, two ergosterol auxotrophic strains (Dharmacon, Chicago, IL, USA) of *Saccharomyces cerevisiae* that lacked the AACT or HMGS allele, named YPL028W (ΔERG10) and YML126C (ΔERG13), respectively, were used for the experiment. The pYES2 vectors, which contain a yeast galactose-dependent promoter, were used as carriers for target genes in this study. The coding regions of *SaAACT* and *SaHMGS* were amplified with two pairs of primers: pYES2-AACT-F and pYES2-AACT-R, and pYES2-HMGS-F and pYES2-HMGS-R, respectively (Table 3). The forward primers contained the *EcoR* I restriction site, and the reverse primers contained the *Not* I restriction site. The pYES2 vector was digested with *EcoR* I and *Not* I, and then PCR products and the digested fragment of the pYES2 vector were ligated into recombined vector pYES2-SaAACT and pYES2-SaHMGS by the In-Fusion HD Cloning Kit (Clontech, Palo Alto, CA, USA). The two constructed plasmids, pYES2-*SaAACT* and pYES2-*SaHMGS*, were extracted and transformed into YPL028W (ΔERG10) and YML126C (ΔERG13) with the Frozen-EZ Yeast Transformation II Kit (Zymo Research, Irvine, CA, USA). *S. cerevisiae* strain ΔERG10, which lacks the AACT allele, and the *S. cerevisiae* strain ΔERG13, which lacks the HMGS allele, are both haploid yeast strains^59,75^. The transformed heterozygous diploid cells were forced to sporulate on YPG medium (1% yeast extract, 2% bacto-peptone, and 2% galactose)^38^, thereby obtaining viable transformed haploid cells by dissecting tetrads. Haploid transformed cells bearing both the disrupted allele and the plasmid-borne MVA pathway genes were selected on minimal medium SC (-Ura) (6.7% yeast nitrogen base without amino acid, 2% galactose)^37^. The transformed diploid cells were induced to sporulate and subsequently formed haploid cells containing pYES2-*SaAACT* and pYES2-*SaHMGS*. To further observe their growth, transformed haploid strains YPL028W and YML126C were grown separately on YPD (1% yeast extract, 2% bacto-peptone, 2% glucose)^76^ and YPG.

### Expression profiling of *SaAACT* and *SaHMGS* in different tissues and their induction by MeJA

The expression levels of *SaAACT* and *SaHMGS* in different tissues (root, sapwood, heartwood, young leaves, mature leaves, and shoots) and expression profiles after MeJA (Aladdin, Shanghai, China) treatment was detected by qRT-PCR. Two-month-old young seedlings (6–8 leaves) of *S. album* were sprayed with 100 μM MeJA until the leaf surfaces were wet. Samples were then collected at 0 h, 2 h, 6 h, 12 h, 24 h, 48 h and 72 h after treatment. About 1.0 μg of total RNA was reverse transcribed into first-strand cDNA using the PrimeScript RT Reagent Kit (Takara Bio Inc., Dalian, China) according to the manufacturer’s protocols. The reaction was performed by ABI7500 fluorescence quantitative PCR (Applied Biosystems, Thermo Fisher Scientific, Waltham, MA, USA) using the iTaq Universal SYBR Green Supermix as buffer (Applied Biosystems). Primer design, the reaction system and the reaction procedure were performed according to the manufacturer’s instructions. The housekeeping gene, *β-actin*, was selected as the internal control^77^. PCR amplification was performed under the following conditions: 95 °C for 30 s, followed by 35 cycles of 95 °C for 15 s and 60 °C for 60 s. Melting curve analyses were conducted. All experiments were performed in triplicate and the mean value was analyzed. Gene expression analysis using the 2^−ΔΔCT^ method^78^ was used to normalize the relative gene expression of the transcripts in different tissues. Significant differences (*p* < 0.05) were assessed by one-way ANOVA, using Duncan’s multiple range test. The results were represented by different letters.

## Data Availability

All data generated or analyzed during this study are included in this published article and its supplementary information files.
